# The Prevalence of Psychological Status During the COVID-19 Epidemic in China: A Systemic Review and Meta-Analysis

**DOI:** 10.3389/fpsyg.2021.614964

**Published:** 2021-05-04

**Authors:** Wei Li, Huijuan Zhang, Caidi Zhang, Jinjing Luo, Hongyan Wang, Hui Wu, Yikang Zhu, Huiru Cui, Jijun Wang, Hui Li, Zhuoying Zhu, Yifeng Xu, Chunbo Li

**Affiliations:** ^1^Shanghai Key Laboratory of Psychotic Disorders, Shanghai Mental Health Center, Shanghai Jiao Tong University School of Medicine, Shanghai, China; ^2^Shanghai General Hospital, Shanghai Jiao Tong University School of Medicine, Shanghai, China; ^3^Chinese Academy of Science Center for Excellence in Brain Science and Intelligence Technology, Chinese Academy of Science, Shanghai, China; ^4^Brain Science and Technology Research Center, Shanghai Jiao Tong University, Shanghai, China; ^5^Institute of Psychology and Behavioral Science, Shanghai Jiao Tong University, Shanghai, China; ^6^Shanghai Clinical Research Center for Mental Health, Shanghai Mental Health Center, Shanghai Jiao Tong University School of Medicine, Shanghai, China

**Keywords:** mental healthcare, COVID-19 pandemic, meta-analysis, psychological problems, PTSD

## Abstract

The COVID-19 is creating panic among people around the world and is causing a huge public mental health crisis. Large numbers of observational studies focused on the prevalence of psychological problems during the COVID-19 pandemic were published. It is essential to conduct a meta-analysis of the prevalence of different psychological statuses to insight the psychological reactions of general population during the COVID-19 epidemic in China. Sixty six observational studies about the psychological statuses of people during the COVID-19 were included, searching up to 1 December 2020. Strengthening the Reporting of Observational Studies in Epidemiology (STROBE) was used to evaluate the quality of the included studies. OpenMeta[Analyst] was used for the data analysis. High prevalence of acute stress and fear symptoms were observed in the early period of the epidemic. Additionally, anxiety and depression symptoms continued at a high prevalence rate during the epidemic. It should alert the lasting mental health problems and the risk of post-traumatic stress disorder and other mental disorders.

**Systematic Review Registration:** PROSPERO CRD 42020171485.

## Introduction

The coronavirus disease (COVID-19) spread rapidly in China since it first appeared in Wuhan, China, in December 2019 (Liu et al., 2012). The acute respiratory infection caused by severe acute respiratory syndrome coronavirus 2 (SARS-CoV-2) has spread globally due to its high transmission rate (The Novel Coronavirus Pneumonia Emergency Response Epidemiology Team, 2020). On 11 March 2020, the WHO characterized COVID-19 as a pandemic. By 1 October 2020, the cumulative number of infections worldwide has exceeded 36 million, and the number of deaths has exceeded 1 million (World Health Organization, 2020). The COVID-19 is creating panic among people around the world and is causing a public mental health crisis (Dong and Bouey, 2020; Yao et al., 2020).

Looking back at the SARS outbreak in 2003 and the Ebola outbreak in 2014, not only did the incidence of psychological problems such as anxiety, fear, and stress increase during the epidemic period, but the psychological problems were also decelerating the recovery of infected patients (Person et al., 2004; Shultz et al., 2016). In addition, long-term follow-up revealed a significant increase in the incidence of mental disorders such as post-traumatic stress disorder and depression, especially among the health care workers (HCW) and survivors of the infection (Mak et al., 2009; Wu et al., 2009; Liu et al., 2012). Fear of illness and death, social isolation, and reduced income all contribute to the high incidence of mental and psychological problems during the emergence of epidemics (Carvalho et al., 2020). Therefore, targeted intervention according to the prevalence of mental and psychological problems during the epidemic has important social effects.

We conducted a meta-analysis of cross-sectional studies published before 6 March 2020 on the prevalence of different psychological states during early stage of COVID-19 epidemic in China (Li W. et al., 2020). The present study updated the literature retrieval date to 1 December 2020 to search more databases through a more comprehensive retrieval strategy. At the same time, the present study focuses on not only the prevalence of different psychological states, but also the difference of the prevalence among different periods of COVID-19 pandemic. Based on the changes in the epidemic situation and the major events related to the psychological status of people, this study provides an evidence-based data for the prevention and control of the epidemic and psychological crisis intervention in the future.

## Materials and Methods

### Search Strategy

We searched the following databases for studies published before 1 December 2020: PubMed, EMBASE, The Cochrane Library, EBSCO, Web of Science, medRxiv, PsycINFO, Chinese National Knowledge Infrastructure (CNKI), Chongqing VIP database for Chinese Technical Periodicals, WANFANG DATA, Chinese Biological Medical Literature Database, and official information release platform (WeChat Official Account or Weibo). The search terms are described in the Supplementary Material. The reference lists of included articles were hand-checked for further relevant studies, and experts in the field were asked about the ongoing studies.

### Inclusion and Exclusion Criteria

All reports investigating the psychological status during the COVID-19 outbreak were screened using the following inclusion criteria: (a) the survey was carried out by using scales with good reliability and validity, and definite boundary values; (b) information about prevalence, sample size, and time of investigation or time of submission; (c) the survey was conducted after COVID-19 outbreak; (d) the survey was conducted among general population; (e) cross-sectional study; (f) studies published in either English or Chinese. The exclusion criteria were as follows: (a) incomplete outcome data or lack of valid data following contact with the original authors; (b) descriptive studies, qualitative studies, anthropologic studies, review articles, research protocols, case reports, and duplicated reports.

### Screening of Articles and Data Extraction

Three researchers (CD.Z., JJ.L., and HY.W.) independently explored previous studies based on search terms. The retrieved records were managed by Endnote X9. After removing the duplicates, all titles and abstracts of the records were screened by the three independent researchers (CD.Z., JJ.L., and HY.W.), and all studies that could possibly meet the inclusion criteria according to one of the researchers were retrieved as full text. The decision to include or exclude a study was also made by the three independent researchers (CD.Z., JJ.L., and HY.W.). The disagreements were discussed and resolved through discussion with a third reviewer (YK. Z.).

The data were then extracted and checked by two independent reviewers (H.L. and W.L.) using a standardized data collection form. The pertinent data extracted included data source, publication date, sample size, investigation time, population, location, and method of investigation, where possible.

### Quality Assessment of the Studies

The included studies were assessed using the Strengthening the Reporting of Observational Studies in Epidemiology (STROBE) checklist (Vandenbroucke et al., 2007), which includes 22 items for evaluating the title and abstract, introduction, methods, results and discussion, while assigning 1 point for each item, with a total of 22 points.

### Outcome Measures

The primary outcome is the prevalence of different psychological statuses during the COVID-19 outbreak. The secondary outcomes are the prevalence of different psychological statuses in Hubei province and other provinces/cities outside the Hubei province.

### Categorization of Time Periods

According to the dynamic changes in the situation and the major events related to the psychological status (Pan et al., 2020), we divided the epidemic into three time periods: the first period was from 23 January to 1 February 2020, during which the experts announced that the virus could be passed on, the government enforced lockdown in Wuhan, local traffic control and social isolation, and the hospitals faced serious shortages of medical resources and protective materials. The second period was from 2 February to 17 February, 2020, during which the Chinese government dispatched medical teams to Hubei Province for medical assistance, alleviated the shortage of medical resources and protective materials gradually, and set up psychological assistance hotlines in all provinces and cities throughout the country. The third period was from 18 February to 24 April, 2020. During this period, the number of patients recovered and discharged increased, and many provinces and cities down-regulated the level of emergency response to major public health emergencies and psychological medical teams to assist Wuhan.

### Analysis

Meta-analyses were performed using the OpenMeta[Analyst] (Brown University, Rhode Island) (Lau et al., 1992; Viechtbauer, 2010; Wallace et al., 2012). For different psychological statuses, only when no less than five different time points could be extracted from the included studies, a meta-analysis was performed. The studies were listed by the investigation time. The pooled effect size was calculated using the DerSimonian-Laird method for the point at which each new study was chronologically added to the evidence base (Kristian et al., 2011). The forest plots provide a visual representation of the trend of different psychological states with the spread of the epidemic. To present the prevalence of different psychological status during different periods of the COVID-19 epidemic, we performed the subgroup meta-analysis according to different periods.

For each meta-analysis, the heterogeneity was estimated using the inconsistency relative index I^2^, which describes the percentage of variation among studies by heterogeneity and not by chance. Values of I^2^ above 25, 50, and 75% were defined as low, moderate, and high heterogeneity, respectively (Higgins et al., 2011). Because the heterogeneity was high (I^2^ > 75%), we used the random effects model and the DerSimonian-Laird method to interpolate the prevalence with a 95% confidence interval (CI) (Kristian et al., 2011). To identify the potential impact of small sample size (<500), sensitivity analyses were performed.

## Results

### Characteristics of the Included Studies

**Figure 1:**
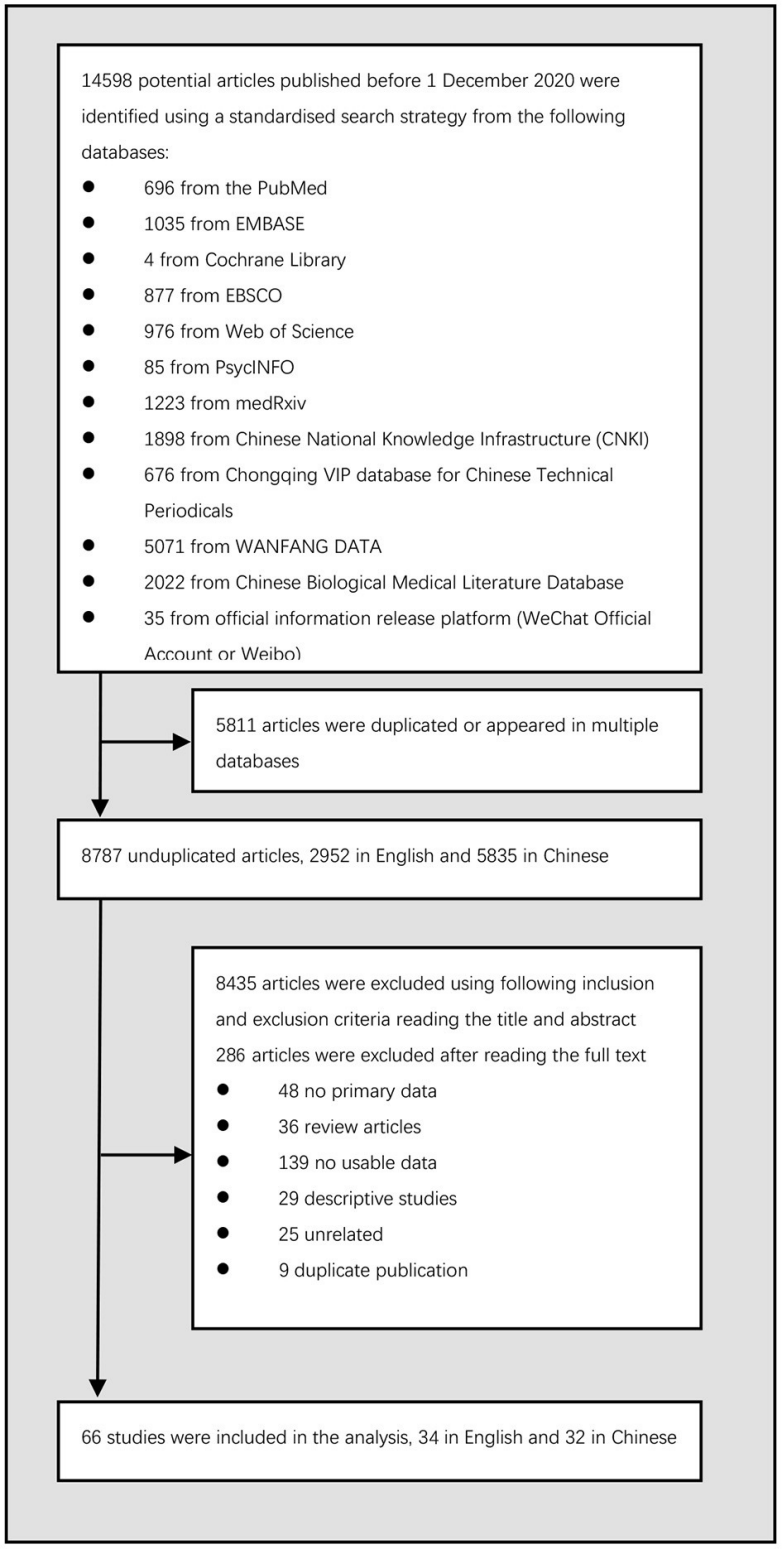
Identification of included studies.

The process of identification of studies included in the analysis was shown in Figure 1. We found a total of 14,598 references in the databases. After removing these duplicates and studies that were reported in more than one article, 8,787 unduplicated articles remained. After reading the title and abstract of these unduplicated articles, we identified 8,435 articles that did not meet our inclusion and exclusion criteria, and after reading the full text, we identified an additional 286 articles that did not meet our criteria. This left us with 66 articles. Among these 66 studies, 34 in English and 32 in Chinese, were included in the subsequent analyses.

The characteristics of these 66 studies are shown in Table 1.

**Table 1:** Characteristics of the included studies.

**No**.	**Study**	**Time of investigation**	**Age (Mean ± SD)**	**Sex (M/F)**	**Location of investigation**	**Questionnaires**	**Sample size**
1	Cai et al., 2020	1/31–2/4	Unavailable	7404/14898	China	Self-compiled questionnaire	22,302
2	Cao H. et al., 2020	2/6–2/13	Unavailable	478/1022	China	HAMA/HAMD	1,500
3	Cao Y. et al., 2020	5/2–5/10	Unavailable	127/303	Shanghai	IES	430
4	Deng et al., 2020	2/13–2/16	32.48 ± 9.05	226/254	China	SAS/SDS/SRQ	480
5	Deng and Lei, 2020	3/2–3/9	Unavailable	77/496	Guangdong province	SAS	573
6	Dong et al., 2020	2/16–2/22	34 ± 9	378/567	China	PHQ-9	945
7	Feng et al., 2020	2/17–3/10	Unavailable	Unavailable	China	SAS/SDS/AIS/PCL-C	53,427
8	Fu et al., 2020	2/18–2/28	Unavailable	376/866	Wuhan	GAD-7/PHQ-9/AIS	1,242
9	Gao et al., 2020	1/31–2/2	32.3 ± 10.0	1560/3267	China	WHO-5/GAD-7	4,827
10	Guo F. et al., 2020	2/18–2/22	Unavailable	15034/11683	China	CES-D/GAD-2	26,717
11	Guo L. et al., 2020	2/3–2/14	Unavailable	3903/9919	China	SCL-90/SASRQ	13,822
12	Guo Y. et al., 2020	2/26–/29	34.4 ± 11.1	1024/1307	China	HADS	2,331
13	He et al., 2020	2/17–2/27	Unavailable	246/876	China	ISI	1,066
14	Huang et al., 2020	2/10–2/15	Unavailable	2676/3585	China	PHQ-9/SAS	6,261
15	Huang and Zhao, 2020	2/3–2/17	35.3 ± 5.6	3284/3952	China	GAD-7/CES-D/PSQI	7,236
16	Huo et al., 2020	2/9–2/14	Unavailable	434/496	Hubei and Yunnan province	GAD-7/PHQ-9	930
17	Jiang et al., 2020a	1/31–2/2	39.6 ± 12.1	261/825	China	Self-compiled questionnaire	1,086
18	Jiang et al., 2020b	2/23–2/29	34.66 ± 12.02	25781/34418	China	SDS/SAI	60,199
19	Li S. et al., 2020	2/16–2/23	Unavailable	833/2168	China	GAD-7/PHQ-9	3,001
20	Li Y. et al., 2020	1/30–2/1	33.2 ± 8.6	209/768	China	GAD-7/PHQ-9	977
21	Liang et al., 2020	1/30	Unavailable	223/361	China	PCL-C	584
22	Lin G. et al., 2020	1/31–2/8	27.7 ± 10.9	213/591	Hainan province	Self-compiled questionnaire	804
23	Lin L. et al., 2020	2/5–2/10	Unavailable	Unavailable	China	GAD-7/PHQ-9/ASDS	3,826
24	Lin L.-Y. et al., 2020	2/5–2/27	Unavailable	1685/3956	China	GAD-7 /PHQ-9/ASDS/ISI	5,641
25	Lin Y. et al., 2020	1/24–2/24	Unavailable	733/1713	China	STAI	2,446
26	Liu et al., 2020	1/30–2/3	Unavailable	251/357	China	STAI/SDS/SCL-90	608
27	Liu Y. et al., 2020	2/13–3/4	Unavailable	301/461	China	SCL-90	762
28	Liu Z. et al., 2020	3/11–3/15	Unavailable	224/503	Guangdong province	GAD-7/PHQ-9	727
29	Luo F. et al., 2020	3/14–3/17	45.0 ± 10.0	122/361	Hubei province	SAS/SDS	483
30	Qi et al., 2020	2/25–3/15	31.8 ± 8.6	250/395	China	PSS-10	645
31	Qiu et al., 2020	1/31–2/10	Unavailable	Unavailable	China	Self-compiled questionnaire	52,730
32	Ran et al., 2020	2/23–3/2	28.7 ± 10.64	586/1184	China	GAD-7 /PHQ-9/PHQ-15	1,770
33	Ren Y. et al., 2020	2/14–3/29	Unavailable	360/812	China	GAD-7/PHQ-9/SCL-90/PSS-10/ISI/PCL-5	1,172
34	Ren Z. et al., 2020	2/9–2/20	Unavailable	2030/4100	China	GAD-7/PHQ-9	6,130
35	Shi et al., 2020	2/28–3/11	35.97 ± 8.22	27149/29530	China	GAD-7/PHQ-9/ISI/ASDS	56,679
36	Song F. et al., 2020	1/28–2/20	Unavailable	553/525	China	SCL-90	1,078
37	Song L. et al., 2020	4/9–4/22	35.35 ± 6.61	183/526	China	GAD-7/CES-D/ISI	709
38	Sun et al., 2021	1/30–2/3	Unavailable	Unavailable	China	PCL-5	2,091
39	Sun M. et al., 2020	1/28–2/4	Unavailable	323/887	China	GAD-7	3,111
40	Sun Q. et al., 2020	2/5–2/19	Unavailable	1162/1972	Except for Hubei province	GAD-7 /PHQ-9/ISI	3,134
41	Tan et al., 2020	2/24–2/25	30.8 ± 7.4	501/172	Chongqing	IES-R/DASS-21/ISI	673
42	Tian et al., 2020	1/31–2/2	35.01 ± 12.8	549/511	China	SCL-90	1,060
43	Wang C. et al., 2020	1/31–2/2	Unavailable	396/814	China	IES-R/DASS	1,210
44	Wang J. et al., 2020	2/4–2/18	Unavailable	2824/3613	China	PSQI	6,437
45	Wang M. et al., 2020	2/1–2/18	Unavailable	576/925	China	GAD-7/PHQ-9/SRQ-20/ISI	1,501
46	Wang et al., 2020a	1/31–2/2	32.32 ± 9.98	1560/3267	China	GAD-7/WHO-5	4,827
47	Wang et al., 2020b	2/20–2/22	Unavailable	406/623	China	SAS/SDS	1,029
48	Wu M. et al., 2020	2/13–2/29	Unavailable	13304/11485	China	HADS	24,789
49	Xiao et al., 2020	2/1–3/31	25.05 ± 9.18	1037/2038	China	GAD-7/PHQ-9	3,075
50	Yang B. et al., 2020	2/2–2/3	Unavailable	213/414	Sichuan province	GAD-7/PHQ-9	627
51	Yang L. et al., 2020	2/1–2/9	Unavailable	142/379	Fujian province	PQEEPH	521
52	Yang S. et al., 2020	3/5–3/14	Unavailable	1239/1196	Deqing and Taizhou	GAD-7/PHQ-9	2,435
53	Yang T. et al., 2020	2/13–2/15	Unavailable	185/148	Wuhan	GAD-7/PHQ-9	333
54	Yang X. et al., 2020	2/1–2/4	33.84 ± 12.28	542/1096	China	PSS	1,638
55	Yang Y. et al., 2020	2/19–2/21	Unavailable	1548/1611	China	GHQ-20	3,159
56	Yu et al., 2020	2/17–2/27	Unavailable	1180/1847	Enshi	SAS	3,027
57	Zhang J. et al., 2020	2/10–2/15	36.45 ± 2.14	0/300	Changzhi	SCL-90	300
58	Zhang et al., 2020b	2/1–2/5	Unavailable	617/561	Wuhan	ISI	1,178
59	Zhao et al., 2020	2/18–2/25	29.17 ± 10.58	Unavailable	China	PSQI	1,722
60	Zhen and Zhou, 2020	1/27–1/30	Unavailable	361/689	China	Self-compiled questionnaire	1,050
61	Zhong et al., 2020	2/13–2/24	Unavailable	5685/10363	China	SASRQ	16,048
62	Zhou and Liu, 2020	3/2–3/5	33.22 ± 0.61	73/138	Hubei province	PQEEPH	211
63	Zhu et al., 2020b	2/5–2/7	33 ± 9	380/996	China	SAS/SDS	1,376
64	Zhu et al., 2020a	2/19–2/26	Unavailable	424/568	China	SAS	992
65	Zhu X. et al., 2020	1/30–2/13	Unavailable	2176/4219	China	GAD-7/PHQ-9/SRQ-20	63,85
66	Zhu Z. et al., 2020	2/17–3/10	Unavailable	410/512	China	SCL-90	922

*HAMA, Hamilton Anxiety Scale; HAMD, Hamilton Depression Scale; IES, Impact of Event Scale; SAS, Self-rating Anxiety Scale; SDS, Self-rating Depression Scale; SRQ, Stress Response Questionnaire; PHQ-9, 9-item Patient Health Questionnaire; AIS, Athens Insomnia Scale; PLC-C, Post-traumatic Stress Disorder Checklist-Civilian Version; GAD-7, 7-item anxiety scale; CES-D, Center for Epidemiological Survey, Depression Scale; GAD-2, 2-item anxiety scale; WHO-5, 5-item World Health Organization Well-Being Index; SCL-90, 90-item Symptom Check List; SASRQ, Stanford Acute Stress Reaction Questionnaire; HADS, Hospital Anxiety and Depression Scale; ISI, Insomnia Severity Index; PSQI, Pittsburgh Sleep Quality Index; SAI, State Anxiety Inventory; ASDS, Acute Stress Disorder Scale; STAI, state-trait anxiety inventory; PSS-10, 10-item Perceived Stress Scale; PHQ-15, 15-item Patient Health Questionnaire; IES-R, Impact of Event Scale-Revised; DASS-21, 21-item Depression Anxiety Stress Scale; SRQ-20, 20-item Stress Response Questionnaire; PQEEPH, Psychological Questionnaires for Emergent Events of Public Health; PSS, Perceived Stress Scale; GHQ-20, General Health Questionnaire*.

The respondents of seven studies came from Hubei province (Cao H. et al., 2020; Fu et al., 2020; Huo et al., 2020; Luo F. et al., 2020; Yang T. et al., 2020; Yu et al., 2020; Zhou and Liu, 2020); the respondents of the thirteen studies came from provinces and cities other than Hubei province (Cao H. et al., 2020; Deng and Lei, 2020; Fu et al., 2020; Guo L. et al., 2020; Huo et al., 2020; Lin G. et al., 2020; Liu Z. et al., 2020; Sun Q. et al., 2020; Tan et al., 2020; Yang B. et al., 2020; Yang L. et al., 2020; Yang S. et al., 2020; Zhang J. et al., 2020).

### Quality Assessment of the Included Studies

The STROBE evaluation results of the included studies showed that all of studies had scores >11, the lowest score was 12 (Qiu et al., 2020), and the highest score was 22 (Wang et al., 2020a). The average score was (18.56 ± 1.51), which is at the relatively good level.

### Findings From Meta-Analyses

#### The Prevalence of Different Psychological Statuses During the COVID-19 Epidemic

**Figure 2:**
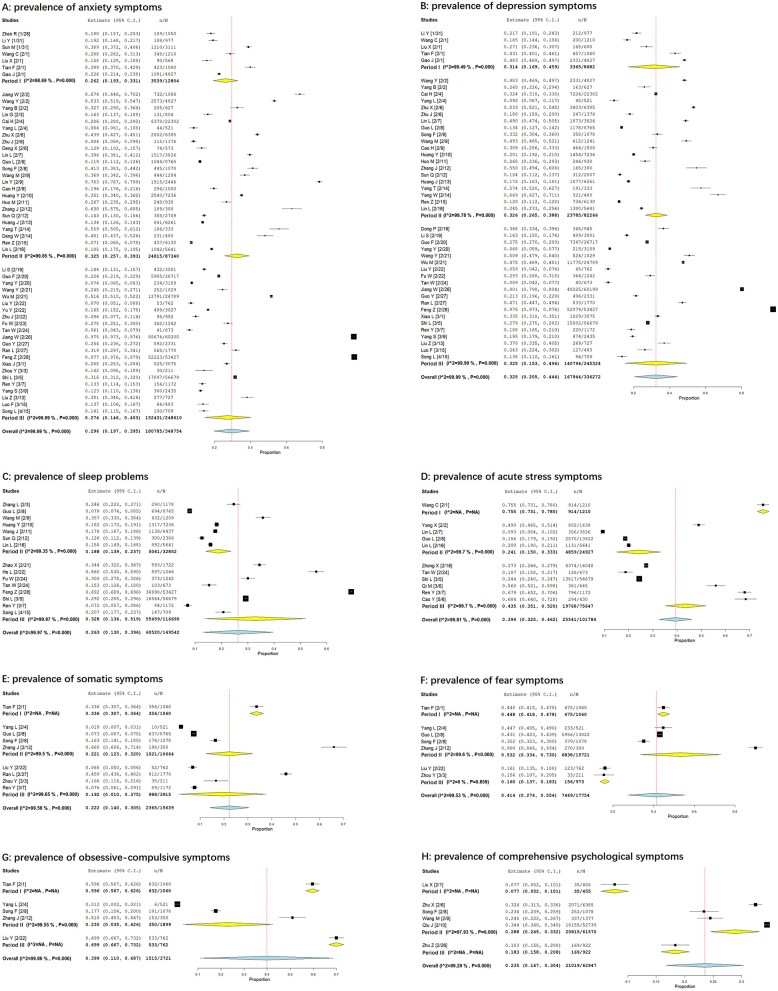
Forest plots: the prevalence of different psychological statuses during the COVID-19 outbreak in China. [**(A)** prevalence of anxiety symptoms; **(B)** prevalence of depression symptoms; **(C)** prevalence of sleep problems; **(D)** prevalence of acute stress symptoms; **(E)** prevalence of somatic symptoms; **(F)** prevalence of fear symptoms; **(G)** prevalence of obsessive-compulsive symptoms; **(H)** prevalence of comprehensive psychological symptoms].

A total of 53 studies investigated the prevalence of anxiety symptoms from 28 January to 15 April, 2020, and the prevalence was found to be 29.6% (95% CI: 19.7–39.5%). There were respectively 7, 24, and 22 studies to investigate the prevalence of anxiety symptoms during three periods of epidemic. The prevalence were found to be 26.2% (95% CI: 19.3–33.1%) in the first period, 32.5% (95% CI: 25.7–39.3%) in the second period, and 27.4% (95% CI: 14.6–40.3%) in the third period of epidemic (see in Figure 2A).

A total of 45 studies investigated the prevalence of depression symptoms from 31 January to 15 April, 2020, with a prevalence of 32.5% (95% CI: 20.5–44.4%). There were respectively 5, 20 and 20 studies to investigate the prevalence of depression symptoms during three periods of epidemic. The prevalence were found to be 31.4% (95% CI: 16.9–45.9%) in the first period, 32.6% (95% CI: 26.5–38.8%) in the second period, and 32.5% (95% CI: 15.3–49.6%) in the third period of epidemic (see in Figure 2B).

A total of 15 studies investigated the prevalence of sleep problems from 3 February to 15 April, 2020, and the overall prevalence was found to be 26.3% (95% CI: 13.0–39.6%). There were respectively seven and eight studies to investigate the prevalence of sleep problems during the second and third period of epidemic. The prevalence were found to be 18.8% (95% CI: 13.9–23.7%) in the second period, and 32.8% (95% CI: 13.6–51.9%) in the third period of epidemic (see in Figure 2C).

A total of 11 studies investigated the prevalence of acute stress symptoms from 1 February to 6 May, 2020, with a prevalence of 39.4% (95% CI: 32.5–46.2%). There were respectively 1, 4, and 6 studies to investigate the prevalence of acute stress symptoms during three periods of epidemic. The prevalence were found to be 75.5% (95% CI: 73.1–78.0%) in the first period, 24.1% (95% CI: 15.0–33.3%) in the second period, and 43.5% (95% CI: 35.1–52.0%) in the third period of epidemic (see in Figure 2D).

A total of nine studies investigated the prevalence of somatic symptoms from 1 February to 7 March, 2020, with a prevalence of 22.2% (95%CI: 14.0–30.5%). There were respectively 1, 4, and 4 studies to investigate the prevalence of somatic symptoms during three periods of epidemic. The prevalence were found to be 33.6% (95% CI: 30.7–36.4%) in the first period, 22.1% (95% CI: 12.3–32.0%) in the second period, and 19.2% (95% CI: 1.0–37.5%) in the third period of epidemic (see in Figure 2E).

A total of seven studies investigated the prevalence of fear symptoms from 1 February to 3 March, 2020, with a total incidence of 41.4% (95% CI: 27.4–55.4%). There were respectively 1, 4, and 2 studies to investigate the prevalence of fear symptoms during three periods of epidemic. The prevalence were found to be 44.8% (95% CI: 41.8–47.8%) in the first period, 53.2% (95% CI: 33.4–73.0%) in the second period, and 16.0% (95% CI: 13.7–18.3%) in the third period of epidemic (see in Figure 2F).

A total of five studies investigated the prevalence of obsessive-compulsive symptoms from 1 February to 22 February, 2020, with a total incidence of 39.9% (95% CI: 11.0–68.7%). There were respectively 1, 3, and 1 studies to investigate the prevalence of obsessive-compulsive symptoms during three periods of epidemic. The prevalence were found to be 59.6% (95% CI: 56.7–62.6%) in the first period, 23.0% (95% CI: 3.5–42.6%) in the second period, and 69.9% (95% CI: 66.7–73.2%) in the third period of epidemic (see in Figure 2G).

A total of six studies did not classify different psychological statuses, but used some comprehensive mental health questionnaires to investigate it from 1 February to 26 February, 2020. The prevalence of comprehensive psychological symptoms was 23.5% (95% CI: 16.7–30.4%). There were respectively 1, 4 and 1 studies to investigate the prevalence of comprehensive psychological symptoms during three periods of epidemic. The prevalence were found to be 7.7% (95% CI: 5.2–10.1%) in the first period, 28.8% (95% CI: 24.5–33.2%) in the second period, and 18.3% (95% CI: 15.8–20.8%) in the third period of epidemic (see in Figure 2H).

#### The Prevalence of Different Psychological Status in Hubei Province and Other Provinces/Cities Outside Hubei Province

**Figure 3:**
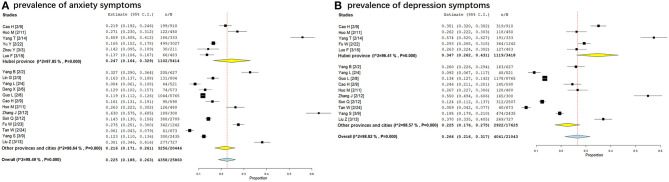
Forest plots: the prevalence of anxiety and depression symptoms in Hubei province and other provinces/cities. [**(A)** prevalence of anxiety symptoms in Hubei province and other provinces/cities; **(B)** prevalence of anxiety symptoms in other provinces/cities outside Hubei province and other provinces/cities].

A total of six studies investigated the prevalence of anxiety symptoms in Hubei province from 9 February to 15 March, 2020, with a prevalence of 24.7% (95% CI: 16.4–32.9%). A total of 13 studies investigated the prevalence of anxiety symptoms in provinces and cities other than Hubei province from 2 February to 13 March, 2020, with a prevalence of 21.6% (95%CI: 17.1–26.1%) (See in Figure 3A).

A total of five studies investigated the prevalence of depression symptoms in Hubei province. The investigation period was from 9 February to 15 March, 2020, with a prevalence of 34.7% (95% CI: 26.2–43.1%). A total of 10 studies conducted investigations on the prevalence of depression symptoms in provinces and cities other than Hubei province, from 2 February to 13 March, 2020, with a prevalence of 22.5% (95%CI: 17.6–27.5%) (see in Figure 3B).

### Sensitivity Analyses

The studies with small sample size (sample size <500) were excluded for sensitivity analysis (Cao Y. et al., 2020; Deng et al., 2020; Luo F. et al., 2020; Yang T. et al., 2020; Zhang J. et al., 2020; Zhou and Liu, 2020). It was found that the results did not change in direction, indicating that the results were relatively stable (Table 2 and S2 in Supplementary Material).

**Table 2:** Sensitivity analysis: the prevalence of different psychological statuses after removing small-sample study.

	**Period 1 (23th Jan−1st Feb)**	**Period 2 (2nd Feb−17th Feb)**	**Period 3 (18th Feb−24th Apr)**	**Overall**
Anxiety symptoms	26.2% (95% CI: 19.3–33.1%)	29.3% (95% CI: 22.0–36.5%)	28.8% (95% CI: 15.4–42.2%)	28.6% (95% CI: 18.2–39.0%)
Depression symptoms	31.4% (95% CI: 16.9–45.9%)	28.0% (95% CI: 21.5–34.4%)	32.8% (95% CI: 15.1–50.4%)	30.6% (95% CI: 18.1–43.1%)
Sleep problems	NA	18.8% (95% CI: 13.9–23.7%)	32.8% (95% CI: 13.6–51.9%)	26.3% (95% CI: 13.0–39.6%)
Acute stress symptoms	75.5% (95% CI: 73.1–78.0%)	24.1% (95% CI: 15.0–33.3%)	38.7% (95% CI: 30.4–46.9%)	36.5% (95% CI: 29.6–43.5%)
Somatic symptoms	33.6% (95% CI: 30.7–36.4%)	8.4% (95% CI: 2.8–14.0%)	20.1% (95% CI: −1.9–42.0%)	17.0% (95% CI: 8.7–25.3%)
Fear symptoms	44.8% (95% CI: 41.8–47.8%)	40.9% (95% CI: 35.5–46.4%)	16.1% (95% CI: 13.5–18.8%)	36.8% (95% CI: 26.4–47.1%)
Obsessive-compulsive symptoms	59.6% (95% CI: 56.7–62.6%)	9.4% (95% CI: −6.8–25.6%)	69.9% (95% CI: 66.7–73.2%)	37.1% (95% CI: 4.8–69.4%)
Comprehensive psychological symptoms	NA	28.8% (95% CI: 24.5–33.2%)	18.3% (95% CI: 15.8–20.8%)	26.7% (95% CI: 21.6–31.8%)
**Anxiety symptoms**				
Hubei province				19.0% (95% CI: 13.8–24.3%)
Other cities/provinces				17.7% (95% CI: 13.8–21.6%)
**Depressive symptoms**				
Hubei province				32.1% (95% CI: 26.5–37.7%)
Other cities/provinces				18.3% (95% CI: 13.8–22.8%)

*NA: There was no study investigated the prevalence of the psychological status during the time period*.

## Discussion

Compared with previous meta-analysis studies focusing on the mental health during the Covid-19 outbreak (Hessami et al., 2020; Luo M. et al., 2020; Ren X. et al., 2020; Wu T. et al., 2020), the present study tried to show psychological statuses during different periods of epidemic through subgroup analysis. By reviewing the psychological conditions at different periods after the occurrence of the stress event of the COVID-19 epidemic, according to the results of our research, more targeted psychological assistance can be arranged at appropriate time point to help people during public emergent events.

An overview of the different psychological statuses during the COVID-19 epidemic in China showed that although the prevalence of acute stress symptoms reached a high level in the early stage of the epidemic, it gradually declined with the progress of the epidemic. However, the prevalence of anxiety and depression symptoms did not improve with the control of the epidemic, but still stayed at a high level, which was significantly higher than the average level of anxiety and depression according to the results from meta-analyses on prevalence of depression and anxiety in Chinese general population before the COVID-19 epidemic (Baxter et al., 2016; Guo et al., 2016; Wang et al., 2017). Previous studies found that anxiety and depression are risk factors for post-traumatic stress disorder (PTSD) (Grekin and O'hara, 2014; Song et al., 2018). Thus, the continued high prevalence of anxiety and depression symptoms during an epidemic may account for the elevated risk of long-term psychological problems (such as PTSD). Timely intervention for anxiety and depression during the epidemic is also helpful in preventing from the incidence of PTSD and related mental disorders.

In the early period of the COVID-19 epidemic, the public's response to the epidemic was not only reflected in the unknown pathogenic capacity and lethality of the virus, but also in the trust in the national public health response capacity and the effectiveness of personal protection measures (Dong and Bouey, 2020). Furthermore, with the promulgation of public health policies, such as the lockdown of the city, the blocking of traffic, and social isolation, the public's fear of COVID-19 increased (Wu et al., 2009). Therefore, the prevalence of fear and acute stress symptoms, the two acute psychological reactions to traumatic events, which quickly increased at the early period, and the prevalence was significantly higher than other psychological problems (Prati et al., 2012; Santos-Reyes and Gouzeva, 2020). Under the intervention of epidemic prevention and control at the national level, the prevalence of fear and acute stress symptoms decreased at the late period of epidemic.

Previous studies on the psychological reaction of the public during COVID-19 mentioned the “Psychological Typhoon Eye” effect (Yáñez et al., 2020; Zhang et al., 2020,a; Zhang S. X. et al., 2020). At the beginning of the epidemic, the residents in Hubei province did not realize the severity of the epidemic and felt that the virus was far away from them. The Hubei Provincial Government did not take strong measures in time. The information received by people is not symmetrical with the facts, it will cause greater panic later. This sent a false signal to the people: this new disease is not serious and can be prevented and controlled. Thus, the true situation of the epidemic was concealed. Furthermore, the residents outside the Hubei province appeared to be more anxious due to the asymmetry of information, and the media reported that the epidemic was very serious (Zhang et al., 2020a). This study did not found that the prevalence of anxiety and depression symptoms outside Hubei province were significantly higher than the prevalence inside Hubei province. However, the results of sensitivity analysis showed the prevalence of depression symptoms inside Hubei province is higher than the prevalence outside Hubei province. This may be related to the explosive increase of infected cases in Hubei province at the early stage of the epidemic, but the local government did not take active and effective measures to prevent the epidemic. However, few studies have been carried out on the prevalence of psychological statuses of residents in Hubei Province, which may be one of the reasons for the insignificant typhoon eye effect. Further researches are needed to show the effect in the future.

## Limitations

However, the study had several limitations. Firstly, although we have tried to avoid the influence of noise on the results, some confounding factors may still influence the results. In order to reduce the impact of noise on the results, we used more stringent inclusion criteria. Therefore, the present study only focused studies conducted in general population, the study population may be more homogeneous, which may partly reduce the influence of possible noise. At the same time, all of the included studies were conducted quality assessment and were at the relatively good level. Additionally, in the sensitivity analysis, when we excluded the studies with small sample size to redo meta-analysis. It was found that the results did not change in direction, indicating that the results were relatively stable. For the longitudinal observation of the dynamic psychological status, the optimal way is to conduct a long-term cross-sectional survey of a specific population through systematic sampling. However, during the epidemic, it was difficult to restrict the population of investigation through an online survey. Additionally, the results of this current study show that there is significant heterogeneity among the studies. The heterogeneity is still large after subgroup analysis, which may be due to the fact that the included studies investigated very different population and settings.

## Conclusions

There are different characteristics of the prevalence of psychological problems/symptoms during the COVID-19 epidemic. The persistently high prevalence of anxiety and depression symptoms during the epidemic could be a risk factor for PTSD and other mental disorders after the outbreak. Therefore, timely implementation of mental health policies is urgently needed for the public mental health crisis during the fight against COVID-19.

## Data Availability Statement

The original contributions presented in the study are included in the article/Supplementary Material, further inquiries can be directed to the corresponding author/s.

## Author Contributions

CL and HL designed the study. WL, HL, and HZ were responsible for drafting the research searching strategy and data extraction. JL, CZ, YZ, and HW conducted the searching and screening of studies. WL drafted the manuscript. HL, HW, HC, JW ZZ, YX, and CL made critical revisions. All authors approved the final version for publication.

## Conflict of Interest

The authors declare that the research was conducted in the absence of any commercial or financial relationships that could be construed as a potential conflict of interest.
