# Supplementary material for: The Prevalence of Psychological Status During the COVID-19 Epidemic in China: A Systemic Review and Meta-Analysis
Source: Front Psychol. 2021 May 4;12:614964. doi: 10.3389/fpsyg.2021.614964 (PMC8129549; doi:10.3389/fpsyg.2021.614964)
Supplement: Supplementary file 1 [file Data_Sheet_1.docx]

**Supplementary materials**

S1. Search terms

- Pubmed:

#5 Search (#4) AND #3 Filters: Publication date from 2020/01/01 to 2020/12/01

#4 Search (((((((mental[Title/Abstract]) OR psycho*[Title/Abstract]) OR depress*[Title/Abstract]) OR anxiety[Title/Abstract]) OR stress[Title/Abstract]) OR trauma*[Title/Abstract]) OR insomnia*[Title/Abstract]) OR sleep[Title/Abstract]

#3 Search (#1) OR #2

#2 Search ((((“COVID-19”[Title/Abstract]) OR “2019-nCoV”[Title/Abstract]) OR "novel coronavirus"[Title/Abstract]) OR “coronavirus disease-2019”[Title/Abstract]) OR “coronavirus disease-19”[Title/Abstract]

#1 Search (wuhan[Title/Abstract]) AND coronavirus[Title/Abstract]

- Embase:

#6 #5 AND 2020:py

#5 #2 AND #4

#4 #1 OR #3

#3 wuhan:ab,ti AND coronavirus:ab,ti

#2 mental:ab,ti OR psycho*:ab,ti OR depress*:ab,ti OR anxiety:ab,ti OR stress:ab,ti OR trauma*:ab,ti OR insomnia*:ab,ti OR sleep:ab,ti

#1 'covid-19':ab,ti OR '2019-ncov':ab,ti OR 'novel coronavirus':ab,ti OR 'coronavirus disease-2019':ab,ti OR 'coronavirus disease-19':ab,ti

- Web of science:

#6 #4 AND #3

Refined by: PUBLICATION YEARS: ( 2020 )

Databases = WOS, BIOSIS, CSCD, DIIDW, INSPEC, KJD, MEDLINE, RSCI, SCIELO

Timespan: All years

Search language=Auto

#5 #4 AND #3

Databases = WOS, BIOSIS, CSCD, DIIDW, INSPEC, KJD, MEDLINE, RSCI, SCIELO

Timespan: All years

Search language=Auto

#4 TOPIC: (mental) OR TOPIC: (psycho*) OR TOPIC: (depress*) OR TOPIC: (anxiety) OR TOPIC: (stress) OR TOPIC: (trauma*) OR TOPIC: (insomnia*) OR TOPIC: (sleep)

Databases = WOS, BIOSIS, CSCD, DIIDW, INSPEC, KJD, MEDLINE, RSCI, SCIELO

Timespan: All years

Search language=Auto

#3 #2 OR #1

Databases = WOS, BIOSIS, CSCD, DIIDW, INSPEC, KJD, MEDLINE, RSCI, SCIELO

Timespan: All years

Search language=Auto

#2 TOPIC: ("COVID-19") OR TOPIC: ("2019-nCoV") OR TOPIC: ("novel coronavirus") OR TOPIC: (“coronavirus disease-2019”) OR TOPIC: (“coronavirus disease-19”)

Databases = WOS, BIOSIS, CSCD, DIIDW, INSPEC, KJD, MEDLINE, RSCI, SCIELO

Timespan: All years

Search language=Auto

#1 TOPIC: (wuhan) AND TOPIC: (coronavirus)

Databases = WOS, BIOSIS, CSCD, DIIDW, INSPEC, KJD, MEDLINE, RSCI, SCIELO

Timespan: All years

Search language=Auto

- EBSCO:

S5 S3 AND S4 Limiters - Published Date: 20200101-20201201

S4 AB mental OR AB psycho* OR AB depress* OR AB anxiety OR AB stress OR AB trauma* OR AB insomnia* OR AB sleep

S3 S1 OR S2

S2 AB wuhan AND AB coronavirus

S1 AB "COVID-19" OR AB "2019-nCoV" OR AB "novel coronavirus" OR AB "coronavirus disease-2019" OR AB "coronavirus disease-19"

- PsycINFO：

S5 S3 AND S4 Limiters - Published Date: 20200101-20201201

S4 AB mental OR AB psycho* OR AB depress* OR AB anxiety OR AB stress OR AB trauma* OR AB insomnia* OR AB sleep

S3 S1 OR S2

S2 AB "COVID-19" OR AB "2019-nCoV" OR AB "novel coronavirus" OR AB "coronavirus disease-2019" OR AB "coronavirus disease-19"

S1 AB wuhan AND AB coronavirus

- Cochrane Library:

#7 #5 AND #6

#6 #3 OR #4

#5 #1 OR #2

#4 (trauma*):ti,ab,kw OR (insomnia*):ti,ab,kw OR (sleep):ti,ab,kw

#3 (mental):ti,ab,kw OR (psycho*):ti,ab,kw OR (depress*):ti,ab,kw OR (anxiety):ti,ab,kw OR (stress):ti,ab,kw

#2 ("COVID 19"):ti,ab,kw OR ("2019 nCoV"):ti,ab,kw OR ("novel coronavirus"):ti,ab,kw OR ("coronavirus disease 2019"):ti,ab,kw OR ("coronavirus disease 19"):ti,ab,kw (Word variations have been searched)

#1 (wuhan):ti,ab,kw AND (coronavirus):ti,ab,kw

(mental):ti,ab,kw OR (psycho*):ti,ab,kw OR (depress*):ti,ab,kw OR (anxiety):ti,ab,kw OR (stress):ti,ab,kw OR (trauma*):ti,ab,kw OR (insomnia*):ti,ab,kw OR (sleep):ti,ab,kw(trauma*):ti,ab,kw OR (insomnia*):ti,ab,kw OR (sleep):ti,ab,kw

- Other database:

Searched by keywords “novel coronavirus”, “new coronavirus”, “2019-nCoV”, “COVID-19”, “2019 ncov”, “coronavirus disease-2019”, “mental”, “psycho*”, “depress*”, “anxiety*”, “stress”, “trauma*”,“insomnia*” , “sleep”; “新型冠状病毒肺炎”, “新冠病毒”, “新冠肺炎”, “COVID-19”, “2019冠状病毒病”, “2019新型冠状病毒感染”, “2019-nCOV肺炎”, “ *新型冠状病毒* ”, “NCP” , “心理*”, “抑郁”, “焦虑”, “应激”, “危机”, “失眠”, “睡眠障碍” and “创伤”.

S2. Sensitivity analysis: the prevalence of different psychological statuses during the COVID-19 outbreak in China


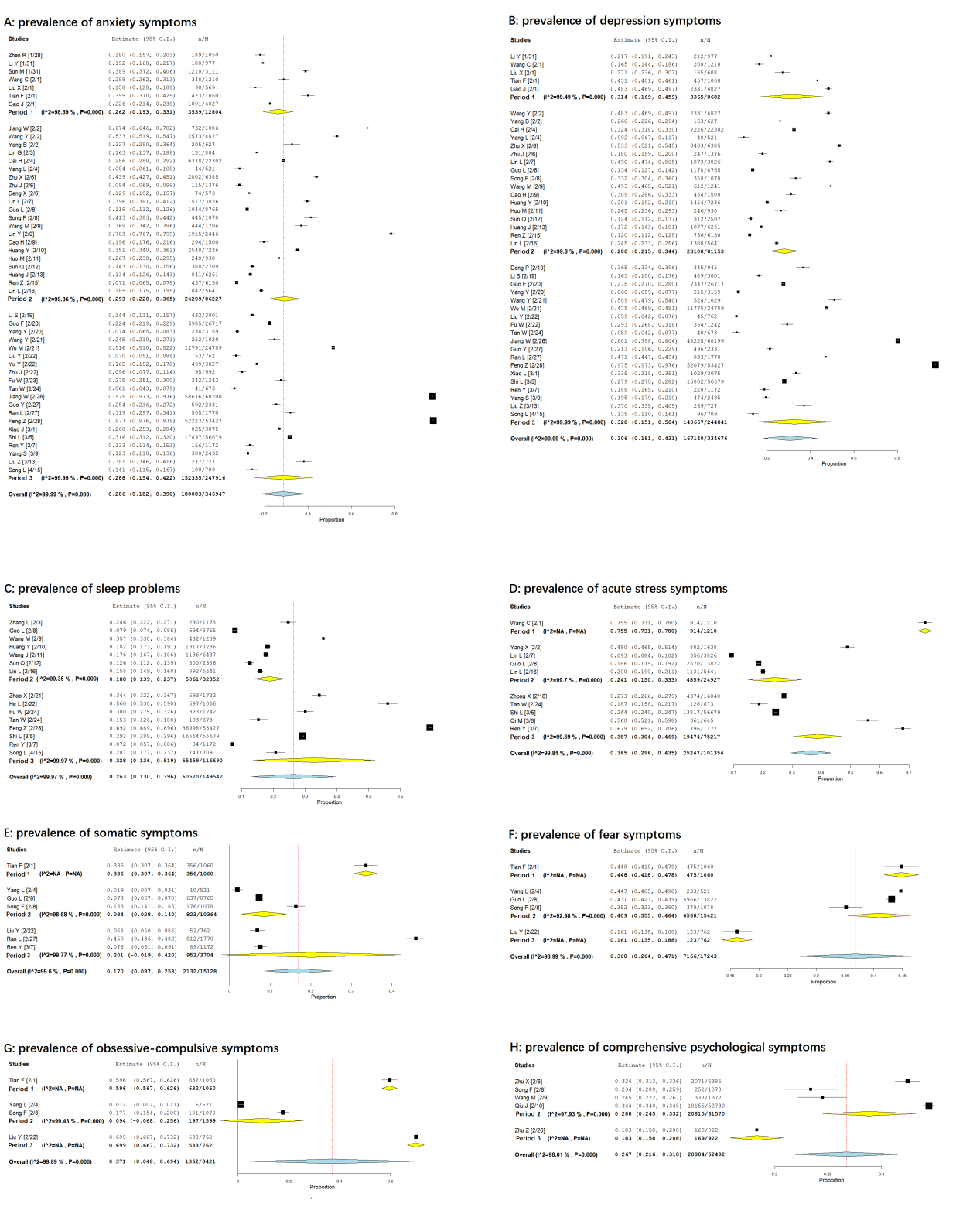


S3. Sensitivity analysis: the prevalence of different psychological statuses in Hubei province and other provinces and cities during the COVID-19 epidemic


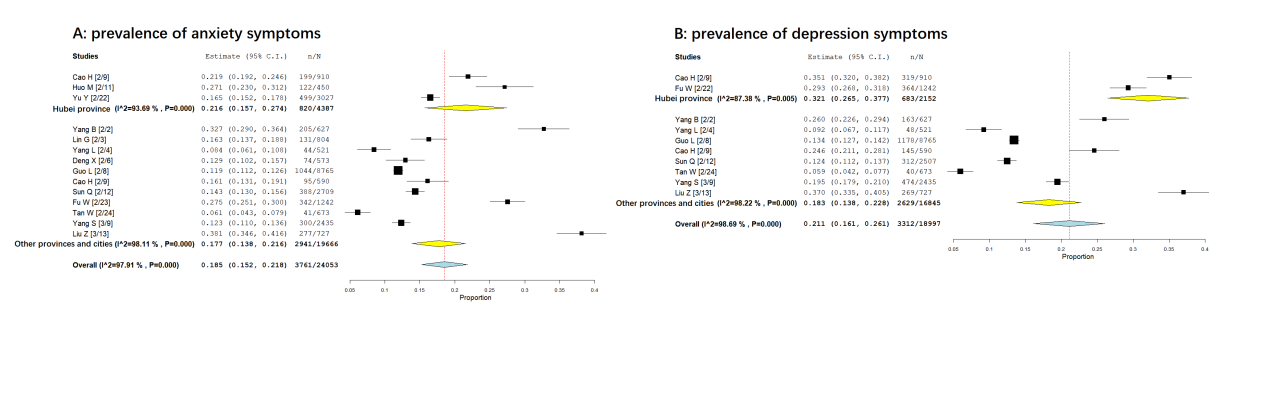


S4. Table of STROBE scores

(on the next page)

| No. | Author | STROBE score | Title and Abstract |  | Introduction | |  | Methods | | | | | | | | |  | Results | | | | |  | Discussion | | | |  | Other |
| --- | --- | --- | --- | --- | --- | --- | --- | --- | --- | --- | --- | --- | --- | --- | --- | --- | --- | --- | --- | --- | --- | --- | --- | --- | --- | --- | --- | --- | --- |
|  |  |  |  |  | Background | Objectives |  | Study design | Setting | Participants | Variables | Data sources/measurement | Bias | Study size | Quantitative variables | Statistical methods |  | Participants | Descriptive data | Outcome data | Main results | Other analyses |  | Key results | Interpretation | Limitations | Generalizability |  | Funding |
| 1 | Cai H | 16 | + |  | + | + |  | + | + | - | + | + | - | - | + | + |  | + | + | + | - | - |  | + | + | + | + |  | - |
| 2 | Cao H | 18 | + |  | + | + |  | - | + | - | + | + | - | - | + | + |  | + | + | + | + | + |  | + | + | + | + |  | + |
| 3 | Cao Y | 19 | + |  | + | + |  | + | + | - | + | + | - | - | + | + |  | + | + | + | + | + |  | + | + | + | + |  | + |
| 4 | Deng W | 18 | + |  | + | + |  | + | + | - | + | + | - | - | + | + |  | + | + | + | + | + |  | + | + | + | + |  | - |
| 5 | Deng X | 19 | + |  | + | + |  | + | + | + | + | + | - | - | + | + |  | + | + | + | + | + |  | + | + | + | + |  | - |
| 6 | Dong P | 20 | + |  | + | + |  | - | + | + | + | + | - | + | + | + |  | + | + | + | + | + |  | + | + | + | + |  | + |
| 7 | Feng Z | 17 | + |  | + | + |  | - | + | + | + | + | - | - | + | + |  | + | + | + | + | + |  | + | + | - | + |  | - |
| 8 | Fu W | 21 | + |  | + | + |  | + | + | + | + | + | + | - | + | + |  | + | + | + | + | + |  | + | + | + | + |  | + |
| 9 | Guo F | 16 | + |  | + | + |  | - | + | - | + | + | - | - | + | + |  | + | + | + | + | + |  | + | + | - | + |  | - |
| 10 | Guo J | 18 | + |  | + | + |  | - | + | - | + | + | - | - | + | + |  | + | + | + | + | + |  | + | + | + | + |  | + |
| 11 | Guo L | 18 | + |  | + | + |  | - | + | - | + | + | - | - | + | + |  | + | + | + | + | + |  | + | + | + | + |  | + |
| 12 | Guo Y | 21 | + |  | + | + |  | + | + | - | + | + | + | + | + | + |  | + | + | + | + | + |  | + | + | + | + |  | + |
| 13 | He L | 17 | + |  | + | + |  | + | + | - | + | + | - | - | - | - |  | + | + | + | + | + |  | + | + | + | + |  | + |
| 14 | Huang J | 19 | + |  | + | + |  | - | + | - | + | + | - | + | + | + |  | + | + | + | + | + |  | + | + | + | + |  | + |
| 15 | Huang Y | 19 | + |  | + | + |  | - | + | + | + | + | - | - | + | + |  | + | + | + | + | + |  | + | + | + | + |  | + |
| 16 | Huo M | 15 | + |  | - | + |  | - | - | - | + | + | - | - | + | + |  | + | + | + | + | + |  | + | + | - | + |  | + |
| 17 | Jiang Wa | 17 | + |  | + | + |  | + | + | + | + | - | - | - | + | + |  | + | + | + | + | + |  | - | + | + | - |  | + |
| 18 | Jiang Wb | 21 | + |  | + | + |  | + | + | + | + | + | + | - | + | + |  | + | + | + | + | + |  | + | + | + | + |  | + |
| 19 | Li S | 20 | + |  | + | + |  | - | + | + | + | + | + | - | + | + |  | + | + | + | + | + |  | + | + | + | + |  | + |
| 20 | Li Yan | 20 | + |  | + | + |  | - | + | + | + | + | + | + | + | + |  | + | + | + | + | + |  | + | + | + | + |  | - |
| 21 | Liang L | 19 | + |  | + | + |  | + | + | - | + | + | - | - | + | + |  | + | + | + | + | + |  | + | + | + | + |  | + |
| 22 | Lin G | 20 | + |  | + | + |  | + | + | + | + | + | + | - | + | + |  | + | + | + | + | + |  | + | + | - | + |  | + |
| 23 | Lin La | 20 | + |  | + | + |  | + | + | + | + | + | - | - | + | + |  | + | + | + | + | + |  | + | + | + | + |  | + |
| 24 | Lin Lb | 18 | + |  | + | + |  | - | + | - | + | + | - | - | + | + |  | + | + | + | + | + |  | + | + | + | + |  | + |
| 25 | Lin Y | 18 | + |  | + | + |  | - | + | - | + | + | - | - | + | + |  | + | + | + | + | + |  | + | + | + | + |  | + |
| 26 | Liu X | 19 | + |  | + | + |  | + | + | - | + | + | - | - | + | + |  | + | + | + | + | + |  | + | + | + | + |  | + |
| 27 | Liu Y | 19 | + |  | + | + |  | + | + | - | + | + | + | - | + | + |  | + | + | + | + | + |  | + | + | - | + |  | + |
| 28 | Liu Z | 18 | + |  | + | + |  | + | + | - | + | + | + | - | + | + |  | + | + | + | + | + |  | + | + | - | + |  | - |
| 29 | Qi M | 18 | + |  | + | + |  | - | + | - | + | + | - | - | + | + |  | + | + | + | + | + |  | + | + | + | + |  | + |
| 30 | Qiu J | 12 | + |  | + | + |  | + | - | - | + | + | - | - | - | - |  | - | + | + | + | - |  | + | + | - | - |  | + |
| 31 | Ran L | 20 | + |  | + | + |  | + | + | - | + | + | + | - | + | + |  | + | + | + | + | + |  | + | + | + | + |  | + |
| 32 | Ren Y | 19 | + |  | + | + |  | + | + | - | + | + | - | - | + | + |  | + | + | + | + | + |  | + | + | + | + |  | + |
| 33 | Ren Z | 18 | + |  | + | + |  | - | + | - | + | + | - | - | + | + |  | + | + | + | + | + |  | + | + | + | + |  | + |
| 34 | Shi L | 18 | + |  | + | + |  | - | + | - | + | + | - | - | + | + |  | + | + | + | + | + |  | + | + | + | + |  | + |
| 35 | Song F | 18 | + |  | + | + |  | - | + | - | + | + | + | - | + | + |  | + | + | + | + | + |  | + | + | + | + |  | - |
| 36 | Song L | 20 | + |  | + | + |  | + | + | - | + | + | + | - | + | + |  | + | + | + | + | + |  | + | + | + | + |  | + |
| 37 | Sun L | 19 | + |  | + | + |  | + | + | - | + | + | + | - | + | + |  | + | + | + | + | + |  | + | + | + | + |  | - |
| 38 | Sun M | 19 | + |  | + | + |  | - | + | - | + | + | + | - | + | + |  | + | + | + | + | + |  | + | + | + | + |  | + |
| 39 | Sun Q | 20 | + |  | + | + |  | + | + | - | + | + | - | + | + | + |  | + | + | + | + | + |  | + | + | + | + |  | + |
| 40 | Tan W | 19 | + |  | + | + |  | - | + | + | + | + | - | - | + | + |  | + | + | + | + | + |  | + | + | + | + |  | + |
| 41 | Tian F | 18 | + |  | + | + |  | - | + | - | + | + | - | - | + | + |  | + | + | + | + | + |  | + | + | + | + |  | + |
| 42 | Tu F | 20 | + |  | + | + |  | + | + | - | + | + | + | + | + | + |  | + | + | + | + | + |  | + | + | + | + |  | - |
| 43 | Wang C | 19 | + |  | + | + |  | + | + | - | + | + | - | - | + | + |  | + | + | + | + | + |  | + | + | + | + |  | + |
| 44 | Wang J | 18 | + |  | + | + |  | - | + | - | + | + | - | - | + | + |  | + | + | + | + | + |  | + | + | + | + |  | + |
| 45 | Wang M | 19 | + |  | + | + |  | - | + | + | + | + | - | - | + | + |  | + | + | + | + | + |  | + | + | + | + |  | + |
| 46 | Wang Ya | 22 | + |  | + | + |  | + | + | + | + | + | + | + | + | + |  | + | + | + | + | + |  | + | + | + | + |  | + |
| 47 | Wang Yb | 20 | + |  | + | + |  | + | + | - | + | + | - | + | + | + |  | + | + | + | + | + |  | + | + | + | + |  | + |
| 48 | Wu M | 18 | + |  | + | + |  | - | + | - | + | + | - | - | + | + |  | + | + | + | + | + |  | + | + | + | + |  | + |
| 49 | Xiao J | 18 | + |  | + | + |  | - | + | - | + | + | - | - | + | + |  | + | + | + | + | + |  | + | + | + | + |  | + |
| 50 | Yang B | 18 | + |  | + | + |  | - | + | + | + | + | + | - | + | + |  | + | + | + | + | + |  | + | + | - | + |  | - |
| 51 | Yang L | 18 | + |  | + | + |  | - | + | + | + | + | - | - | + | + |  | + | + | + | + | + |  | + | + | + | + |  | - |
| 52 | Yang S | 19 | + |  | + | + |  | - | + | - | + | + | + | - | + | + |  | + | + | + | + | + |  | + | + | + | + |  | + |
| 53 | Yang T | 20 | + |  | + | + |  | + | + | + | + | + | + | - | + | + |  | + | + | + | + | + |  | + | + | + | + |  | - |
| 54 | Yang X | 19 | + |  | + | + |  | - | + | - | + | + | + | - | + | + |  | + | + | + | + | + |  | + | + | + | + |  | + |
| 55 | Yang Y | 19 | + |  | + | + |  | + | + | - | + | + | - | - | + | + |  | + | + | + | + | + |  | + | + | + | + |  | + |
| 56 | Yu Y | 19 | + |  | + | + |  | + | + | + | + | + | - | - | + | + |  | + | + | + | + | + |  | + | + | + | + |  | - |
| 57 | Zhang J | 17 | + |  | + | + |  | - | + | - | + | + | - | - | + | + |  | + | + | + | + | + |  | + | + | + | + |  | - |
| 58 | Zhang L | 18 | + |  | + | + |  | - | + | - | + | + | - | - | + | + |  | + | + | + | + | + |  | + | + | + | + |  | + |
| 59 | Zhao Xb | 18 | + |  | + | + |  | - | + | - | + | + | - | - | + | + |  | + | + | + | + | + |  | + | + | + | + |  | + |
| 60 | Zhen R | 18 | + |  | + | + |  | - | + | - | + | + | - | - | + | + |  | + | + | + | + | + |  | + | + | + | + |  | + |
| 61 | Zhong X | 17 | + |  | + | + |  | - | + | - | + | + | - | - | - | + |  | + | + | + | + | + |  | + | + | + | + |  | + |
| 62 | Zhou Y | 17 | + |  | + | + |  | + | + | - | + | + | - | - | + | + |  | + | + | + | + | + |  | + | + | - | + |  | - |
| 63 | Zhu Ja | 19 | + |  | + | + |  | - | + | + | + | + | + | - | + | + |  | + | + | + | + | + |  | + | + | - | + |  | + |
| 64 | Zhu Jb | 20 | + |  | + | + |  | - | + | - | + | + | + | + | + | + |  | + | + | + | + | + |  | + | + | + | + |  | + |
| 65 | Zhu X | 19 | + |  | + | + |  | - | + | - | + | + | + | - | + | + |  | + | + | + | + | + |  | + | + | + | + |  | + |
| 66 | Zhu Z | 18 | + |  | + | + |  | - | + | - | + | + | - | - | + | + |  | + | + | + | + | + |  | + | + | + | + |  | + |
